# Supplementary material for: Activating Parkin-dependent mitophagy alleviates oxidative stress, apoptosis, and promotes random-pattern skin flaps survival
Source: Commun Biol. 2022 Jun 22;5:616. doi: 10.1038/s42003-022-03556-w (PMC9217959; doi:10.1038/s42003-022-03556-w)

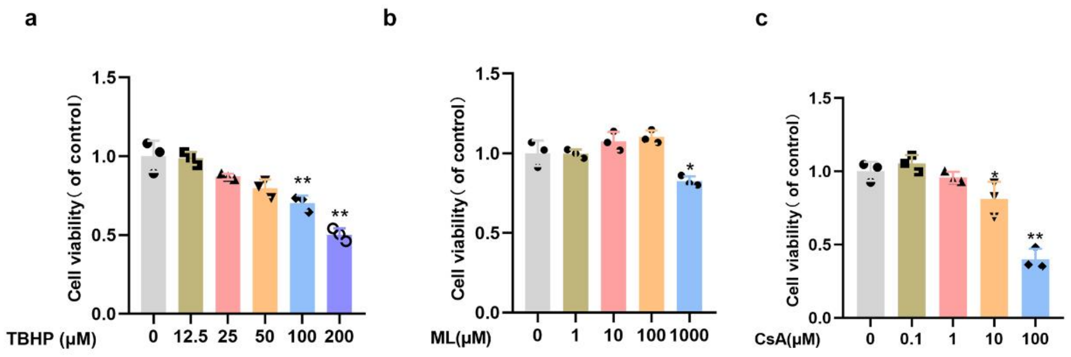


## Supplementary figure 1

**Effects of TBHP, ML, and CsA on the cell viability of HUVECs**

**(a, b, c)** CCK8 assay was performed to determine the cytotoxic effect of various concentrations of TBHP, ML, and CsA on HUVECs for 24 hours. All experiments have been performed at least 3 times. Data are presented as mean ± S.D. n = 3, $^{**}P < 0.01$, $^{*}P < 0.05$.

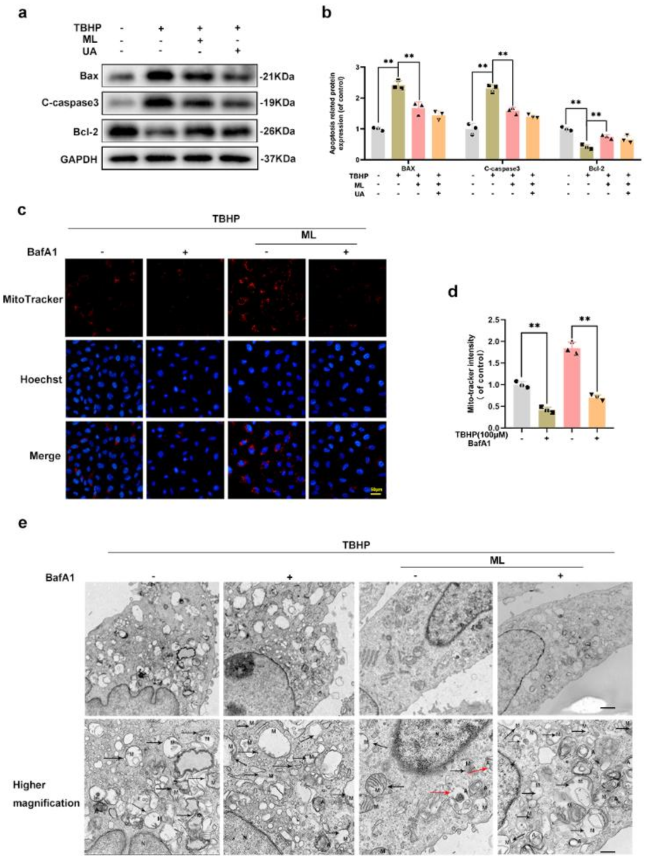


## Supplementary figure 2

### ML upregulated mitophagy and ameliorates TBHP induced apoptosis in HUVECs

**(a, b)** Expression and quantification of protein Bax, C-caspase3, and Bcl-2 were measured by western blot. **(c, d)** A Mito-tracker probe was performed to detect the condition of mitochondrial in the presence and absence of Bafilomycin A1. Then, the fluorescence intensity was quantified (bar: 20 μm). **(e)** Detection of ultrastructure of mitochondria and autophagic change by TEM (×10, 000 or 50, 000) (Black arrow: swollen mitochondria with fractured cristae; Red arrow: autophagolysosome; M: mitochondria; A: autophagosome). All experiments have been performed at least 3 times. Data are presented as mean ± S.D. n = 3, **P < 0.01, *P < 0.05.

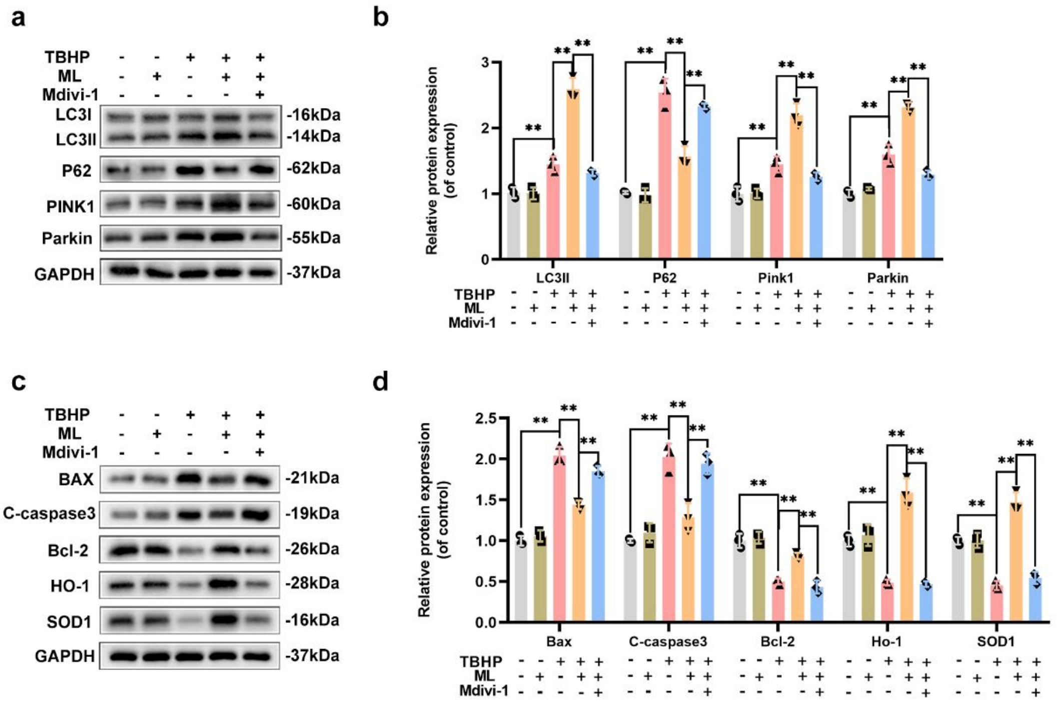


## Supplementary figure 3

**ML protected HUVECs from TBHP induced injury in a mitophagy-dependent manner**

**(a, b)** The protein expression of LC3-II, p62, Pink1, and Parkin in HUVECs treated as above and was quantified and shown by a histogram. **(c, d)** Expression and quantification of apoptosis-related and oxidative-related proteins Bax, C-caspase3, Bcl-2, HO-1, and SOD1 were measured by western blot. All experiments have been performed at least 3 times. Data are presented as mean ± S.D. n = 3, $**P < 0.01$, $*P < 0.05$.

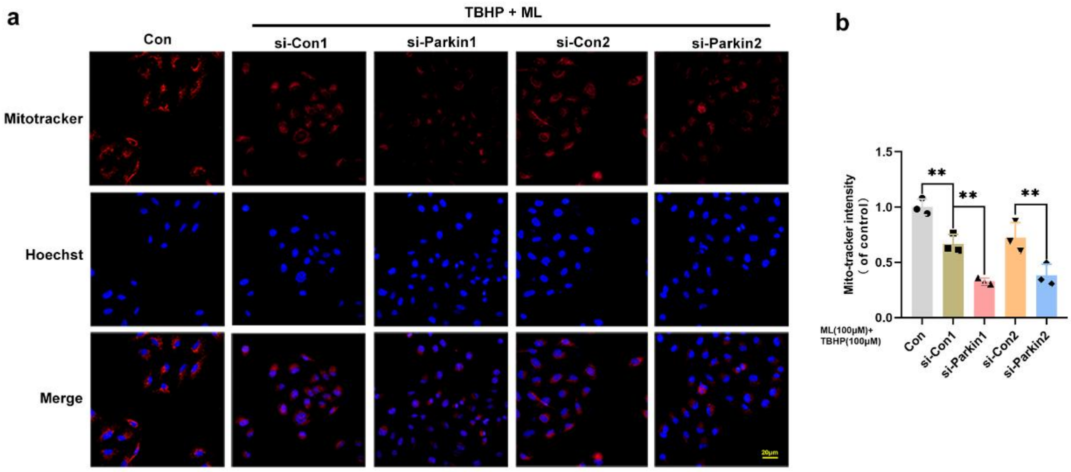


## Supplementary figure 4

**Parkin knockdown decreased the fluorescence intensity of mitochondria in TBHP treated HUVECs**

**(a, b)** A MitoTracker probe was performed to measure fluorescence intensity of mitochondrial after si-RNA transfected. Then, the fluorescence intensity was quantified (bar: 20 μm). All experiments have been performed at least 3 times. Data are presented as mean ± S.D. n = 3, **P < 0.01, *P < 0.05.

# Supplementary figure 5

## Uncropped blot corresponding to Figure 1

Figure1 b

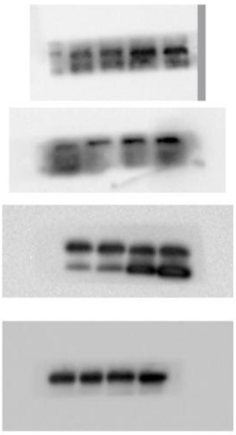

Figure1 d

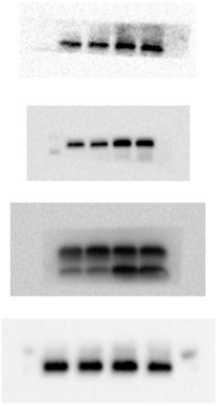

Figure1 f

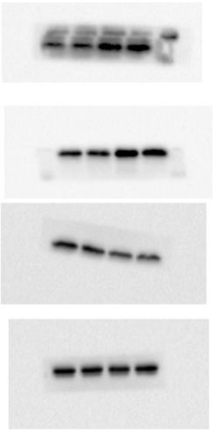

Figure1 k

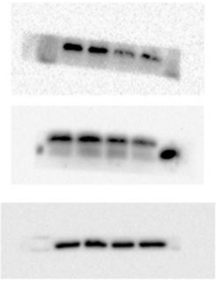

## Uncropped blot corresponding to Figure 2

Figure2-a

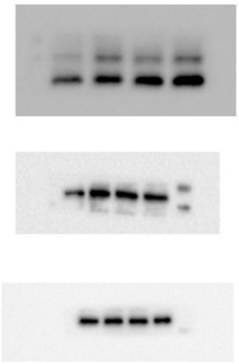

Figure2-g

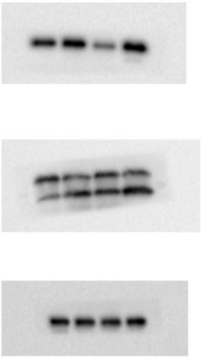

## Uncropped blot corresponding to Figure 3

Figure3-a

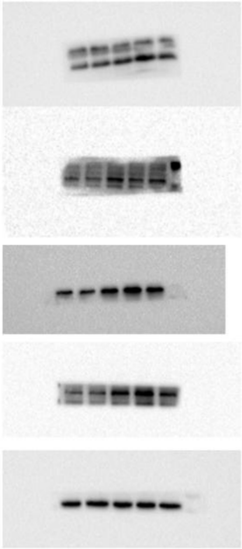

Figure3-c

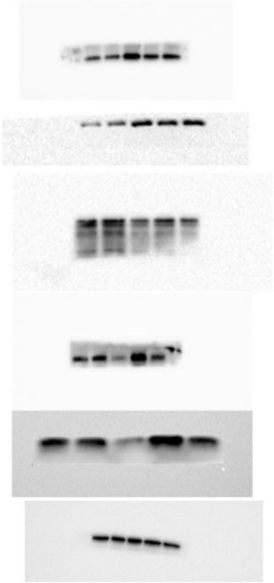

## Uncropped blot corresponding to Figure 4

Figure4-c

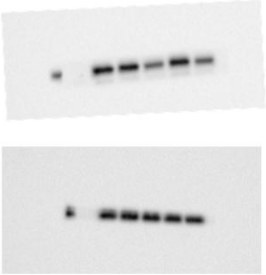

Figure4-e

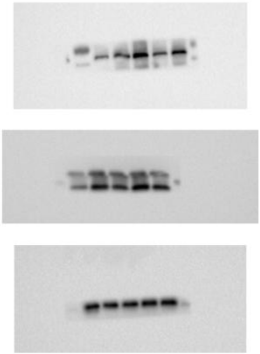

Figure4-g

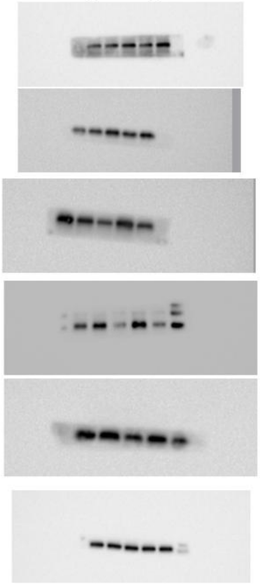

## Uncropped blot corresponding to Figure 5

Figure5-a

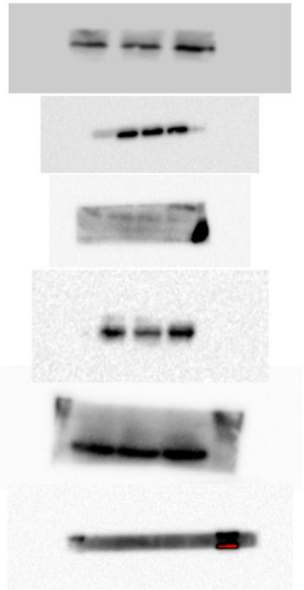

Figure5-e

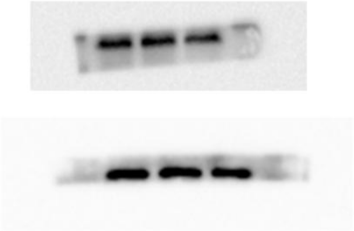

Figure5-g

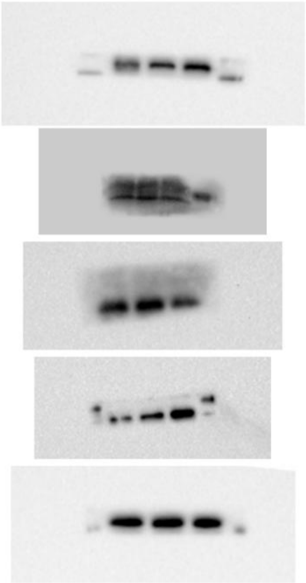

## Uncropped blot corresponding to Figure 6

Figure6-a

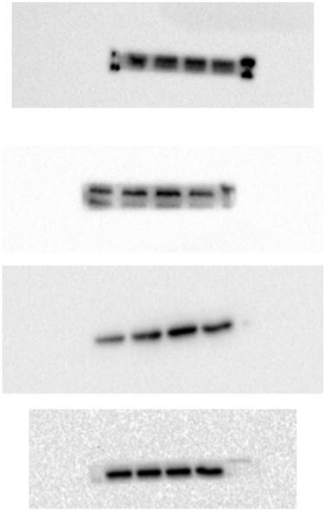

Figure6-c

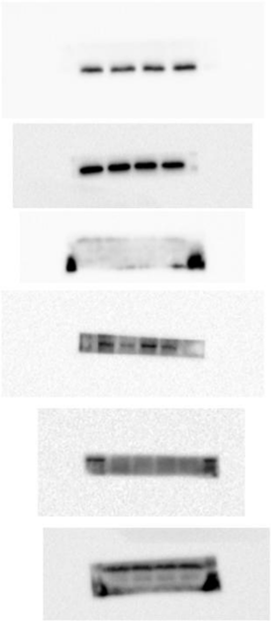

## Uncropped blot corresponding to Supplementary figure 2 and 3

Supplementary Figure 2-a

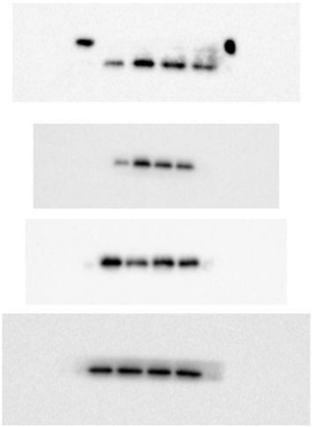

Supplementary Figure 3-a

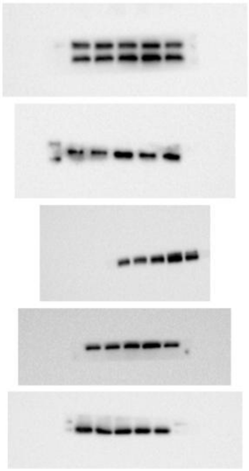

Supplementary Figure 3-c

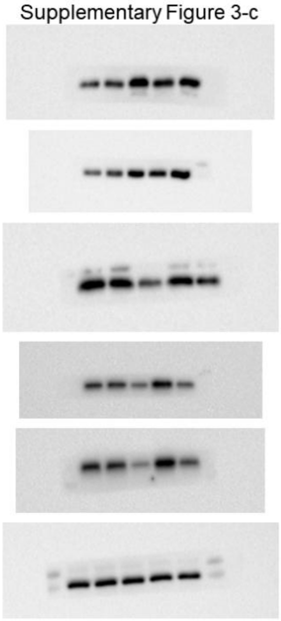

Supplement: Supplementary file 1 — Supplementary Information [file 42003_2022_3556_MOESM1_ESM.pdf]
