# Supplementary material for: Activating Parkin-dependent mitophagy alleviates oxidative stress, apoptosis, and promotes random-pattern skin flaps survival
Source: Commun Biol. 2022 Jun 22;5:616. doi: 10.1038/s42003-022-03556-w (PMC9217959; doi:10.1038/s42003-022-03556-w)
Supplement: Supplementary file 2 — Reporting Summary [file 42003_2022_3556_MOESM2_ESM.pdf]

nature research

Corresponding author(s): DBPR + COMMSBIO-21-1393C

Last updated by author(s): Apr 5, 2022

Reporting Summary

Nature Research wishes to improve the reproducibility of the work that we publish. This form provides structure for consistency and transparency in reporting. For further information on Nature Research policies, see our Editorial Policies and the Editorial Policy Checklist.

Statistics

For all statistical analyses, confirm that the following items are present in the figure legend, table legend, main text, or Methods section.

n/a	Confirmed	
☐	☒	The exact sample size (*n*) for each experimental group/condition, given as a discrete number and unit of measurement
☐	☒	A statement on whether measurements were taken from distinct samples or whether the same sample was measured repeatedly
☐	☒	The statistical test(s) used AND whether they are one- or two-sided<br>*Only common tests should be described solely by name; describe more complex techniques in the Methods section.*
☒	☐	A description of all covariates tested
☒	☐	A description of any assumptions or corrections, such as tests of normality and adjustment for multiple comparisons
☐	☒	A full description of the statistical parameters including central tendency (e.g. means) or other basic estimates (e.g. regression coefficient) AND variation (e.g. standard deviation) or associated estimates of uncertainty (e.g. confidence intervals)
☐	☒	For null hypothesis testing, the test statistic (e.g. *F*, *t*, *r*) with confidence intervals, effect sizes, degrees of freedom and *P* value noted<br>*Give P values as exact values whenever suitable.*
☒	☐	For Bayesian analysis, information on the choice of priors and Markov chain Monte Carlo settings
☒	☐	For hierarchical and complex designs, identification of the appropriate level for tests and full reporting of outcomes
☐	☒	Estimates of effect sizes (e.g. Cohen's *d*, Pearson's *r*), indicating how they were calculated

Our web collection on statistics for biologists contains articles on many of the points above.

Software and code

Policy information about availability of computer code

Data collection	Blood flow was quantified using the perfusion unit and was calculated using the Moor LDI Review software (ver.6.1; Moor Instruments).
Data analysis	Image Lab 3.0 software (Bio-Rad, California, USA) was carried out to quantitate and analyze proteins' grayscale values. The fluorescence intensity was analyzed by Image-Pro Plus 6.0 (Media Cybernetics, MD, USA).

For manuscripts utilizing custom algorithms or software that are central to the research but not yet described in published literature, software must be made available to editors and reviewers. We strongly encourage code deposition in a community repository (e.g. GitHub). See the Nature Research guidelines for submitting code & software for further information.

Data

Policy information about availability of data

All manuscripts must include a data availability statement. This statement should provide the following information, where applicable:

- Accession codes, unique identifiers, or web links for publicly available datasets
- A list of figures that have associated raw data
- A description of any restrictions on data availability

Provide your data availability statement here.

Field-specific reporting

Please select the one below that is the best fit for your research. If you are not sure, read the appropriate sections before making your selection.

☒ Life sciences ☐ Behavioural & social sciences ☐ Ecological, evolutionary & environmental sciences

For a reference copy of the document with all sections, see nature.com/documents/nr-reporting-summary-flat.pdf

Life sciences study design

All studies must disclose on these points even when the disclosure is negative.

Sample size	60 rats were used in this study.
Data exclusions	We excluded a few values with large deviations.
Replication	Each item was were<br>All experiments have been performed at least 3 times independently.
Randomization	In this study 54 rats were randomly numbered from 1 to 54 and all numbers were divided by 3, with the remainder of 1 for Control group (n=18) with remainder of 2 for Melatonin group (n=18) and the rest rats for Melatonin + Compound C group (n=18). And another 6 rats were assigned to the sham group.
Blinding	The data in this study was collected and analyzed by observers who were blinded to the experimental groups.

Reporting for specific materials, systems and methods

We require information from authors about some types of materials, experimental systems and methods used in many studies. Here, indicate whether each material, system or method listed is relevant to your study. If you are not sure if a list item applies to your research, read the appropriate section before selecting a response.

Materials & experimental systems

n/a	Involved in the study	
☐	☒	Antibodies
☐	☒	Eukaryotic cell lines
☒	☐	Palaeontology and archaeology
☐	☒	Animals and other organisms
☒	☐	Human research participants
☒	☐	Clinical data
☒	☐	Dual use research of concern

Methods

n/a	Involved in the study	
☒	☐	ChIP-seq
☒	☐	Flow cytometry
☒	☐	MRI-based neuroimaging

Antibodies

Antibodies used	The following primary antibody were obtained from Proteintech Group (Chicago, IL, USA): Superoxide Dismutase 1 (catalog number:10269-1), GAPDH (catalog number:60004-1), Histone-H3 (catalog number 17168-1), Bcl-2(catalog number:26593-1), Transcription factor EB (catalog number:13372-1) and Bax(catalog number:50599-2). The following primary antibody were obtained from Cell Signaling (Beverly, MA, USA): cleaved caspase 3 (catalog number:9661), Parkin (catalog number:4211), Pink1(catalog number: 6946), AMPK-α (catalog number:5831), and phospho-AMPK-α (catalog number: 2537). The following primary antibody were obtained from Abcam (Cambridge, MA, USA): LC3 (catalog number: ab192890), P62(catalog number: ab207305), and Heme Oxygenase 1(catalog number: ab52947).
Validation	Primary antibody against Superoxide Dismutase 1 has been validated in human, mouse and rat. Applications in IF, WB, IHC and IP. Primary antibody against GAPDH has been validated in human, mouse, rat, yeast and plant. Applications in IF, WB, and IP. Primary antibody against Bcl-2 has been validated in human, mouse and rat. Applications in IF, IHC and WB. Primary antibody against Transcription factor EB has been validated in human, mouse and rat. Applications in IF, WB, IHC and FC. Primary antibody against Bax has been validated in human, mouse and rat. Applications in FC, IP, WB, and ELISA. Primary antibody against Cleaved caspase 3 has been validated in human, mouse, rat and monkey. Applications in FC, IP, WB, and ELISA. Primary antibody against Pink1 has been validated in human and rat. Applications in IP and WB. Primary antibody against Parkin has been validated in human, mouse and rat. Applications in IP and WB. Primary antibody against AMPK-α has been validated in human, mouse and rat. Applications in IP and WB. Primary antibody against phospho-AMPK-α has been validated in human, mouse, rat and monkey. Applications in WB. Primary antibody against Heme Oxygenase 1 has been validated in human and mouse. Applications in WB, Flow Cyt, and IP. Primary antibody against LC3 has been validated in human and mouse. Applications in IHC, WB, and IF. Primary antibody against P62 has been validated in human and rat. Applications in Flow Cyt, IHC-P, WB, ICC/IF, and IP.

Eukaryotic cell lines

Policy information about cell lines

Cell line source(s)	ATCC (Manassas, VA)
Authentication	ATCC (Manassas, VA)
Mycoplasma contamination	The cell lines tested negative for mycoplasma contamination.
Commonly misidentified lines (See ICLAC register)	Not apply.

Animals and other organisms

Policy information about studies involving animals; ARRIVE guidelines recommended for reporting animal research

Laboratory animals	8-week-old healthy male Sprague Dawley rats
Wild animals	The study did not involve wild animals.
Field-collected samples	We collected the rats flap tissues under the normal conditions at room temperature.
Ethics oversight	The Animal Care and Use Committee of Wenzhou Medical University.

Note that full information on the approval of the study protocol must also be provided in the manuscript.
